# Comparative Analysis of the Fecal Bacterial Microbiota of Wintering Whooper Swans (*Cygnus Cygnus*)

**DOI:** 10.3389/fvets.2021.670645

**Published:** 2021-07-12

**Authors:** Wenxia Wang, Songlin Huang, Liangliang Yang, Guogang Zhang

**Affiliations:** ^1^Research Institute of Forestry Policy and Information, Chinese Academy of Forestry, Beijing, China; ^2^Research Institute of Forest Ecology, Environment and Protection, Chinese Academy of Forestry, Beijing, China; ^3^Key Laboratory of Forest Protection of National Forestry and Grassland Administration, Beijing, China

**Keywords:** wintering whooper swans, bacterial microbiota, conservation, LEfSe analysis, 16S-rRNA gene sequencing

## Abstract

There are many and diverse intestinal microbiota, and they are closely related to various physiological functions of the body. They directly participate in the host's food digestion, nutrient absorption, energy metabolism, immune response, and many other physiological activities and are also related to the occurrence of many diseases. The intestinal microbiota are extremely important for maintaining normal physical health. In order to explore the composition and differences of the intestinal microbiota of whooper swans in different wintering areas, we collected fecal samples of whooper swans in Sanmenxia, Henan, and Rongcheng, Shandong, and we used the Illumina HiSeq platform to perform high-throughput sequencing of bacterial 16S rRNA genes. Comparison between Sanmenxia and Rongcheng showed no significant differences in ACE, Chao 1, Simpson, and Shannon indices (*p* > 0.05). Beta diversity results showed significant differences in bacterial communities between two groups [analysis of similarity (ANOSIM): R = 0.80, *p* = 0.011]. Linear discriminant analysis effect size (LEfSe) analysis showed that at the phylum level, the relative abundance of Actinobacteria was significantly higher in Sanmenxia whooper swans than Rongcheng whooper swans. At the genus level, the amount of *Psychrobacter* and *Carnobacterium* in Sanmenxia was significantly higher in Rongcheng, while the relative abundance *Catellicoccus* and *Lactobacillus* was significantly higher in Rongcheng than in Sanmenxia. This study analyzed the composition, characteristics, and differences of the intestinal microbiota of the whooper swans in different wintering environments and provided theoretical support for further exploring the relationship between the intestinal microbiota of the whooper swans and the external environment. And it played an important role in the overwintering physiology and ecology, population management, and epidemic prevention and control of whooper swans.

## Introduction

Whooper swan (*Cygnus cygnus*) is a large migratory bird of Anatidae *Cygnus*, Anseriformes. It is a national level II key protected wildlife in China and is classified as vulnerable (V) by the “China Red Book of Endangered Animals · Birds.” Whooper swans are widely distributed in China, and many provinces have breeding and wintering sites for whooper swans, such as Sanmenxia in Henan, Rongcheng in Shandong, and Qinghai Lake (1).

Rongcheng Whooper Swan Nature Reserve is one of the largest wintering habitats of whooper swans in the world. In the past 10 years, the population has been maintained at more than 3,000 whooper swans every year, and it has become one of the places with the largest number of whooper swans in winter in the world (2, 3). Whooper swan is the largest bird in the reserve, a representative bird of Anseriformes living in the reserve in winter, and an important indicator species in the swan lake wetland (4). The Sanmenxia Reservoir is a wetland in the middle and lower reaches of the Yellow River and is one of the main wintering places for whooper swans in China (5, 6). In recent years, many whooper swans have come to the Sanmenxia for wintering. Whooper swans have become a representative of the wintering birds in Sanmenxia Swan National Wetland Park.

Food resources are very important for the safe wintering and smooth migration of birds to breeding places (7). During the wintering period, whooper swans do not carry out reproductive activities (8–11). In winter, food resources are relatively scarce and extremely important and are related to whether the whooper swans can survive smoothly during the winter. Therefore, adequate food resources are an important part of protecting whooper swans (11). Whooper swans are omnivorous and partial herbivorous large swimming birds (12). They mainly live in grassy lakes, ponds, rivers, and other areas and eat leaves, roots, stems, and crop seeds of aquatic plants seedlings, while some feed on aquatic insects, snails, and mollusks (5, 7, 13).

Whooper swans have different food resources and habitat environments in different wintering areas, so the intestinal microbiota may also vary accordingly. The intestinal microbiota are large in number and diversity and closely related to various physiological functions of the body. They directly participate in the host's food digestion, nutrient absorption, energy metabolism, immune response, and many other physiological activities (14–17), which is simultaneously associated with the occurrence of multiple diseases (18–20), and they are extremely important for maintaining normal physical health. At present, there is no relevant research on the intestinal microbiota of whooper swans in different wintering places. In this study, we sampled fecal of whooper swans from two different wintering places and combined with the food resources and environment during the winter period to study the composition and differences of the bacterial microbiota of the whooper swans. The analysis and discussion of the bacterial microbiota and the wintering environment are of great significance to the physiological ecology and population management of the whooper swans.

## Materials and Methods

### Ethics Statement

This study was carried out in accordance with the recommendations on animal care and ethics of Research Institute of Forest Ecology, Environment and Protection, Chinese Academy of Forestry. The Ethics Committee of Research Institute of Forest Ecology, Environment and Protection also approved the protocol (Ethics Statement of Molecular biology Study of Whooper Swan 201801). The management authority of Sanmenxia Swan National Wetland Park in Henan and the Swan Lake National Nature Reserve in Rongcheng approved the collection of whooper swan fecal samples.

### Study Objects and Areas

**Figure 1:**
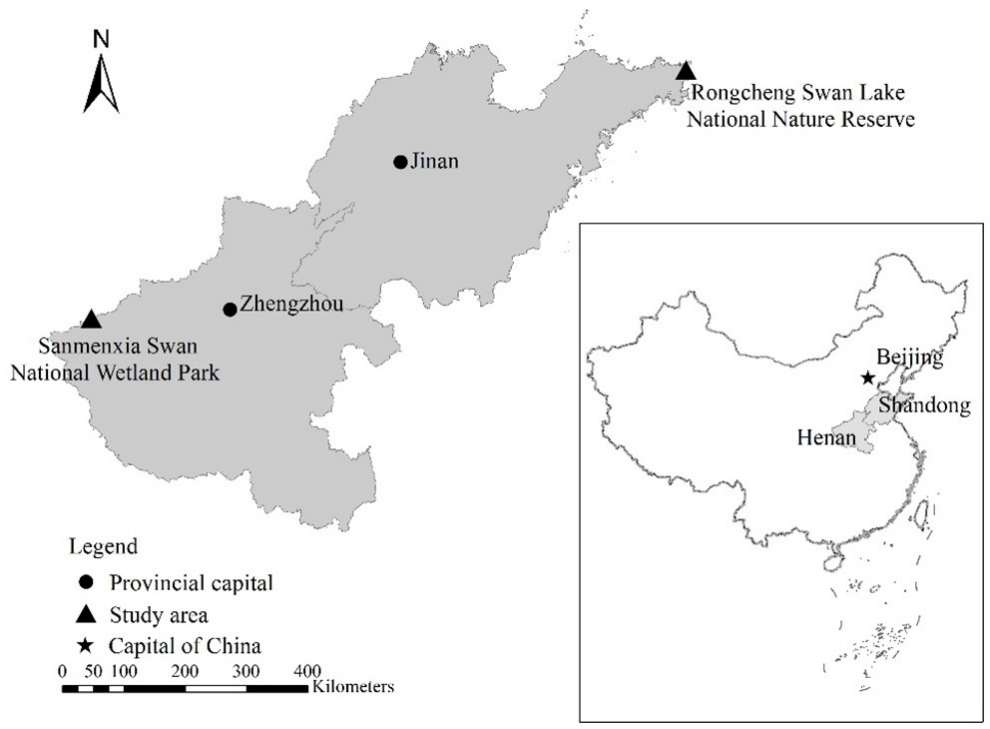
Geographical location of whopper swan's study sites.

The research objects are the whooper swans during the wintering period in Sanmenxia Swan National Wetland Park in Henan and the Swan Lake National Nature Reserve in Shandong Rongcheng (Figure 1). Sanmenxia in Henan and Rongcheng in Shandong are both important wintering sites for whooper swans in northern China.

Henan Sanmenxia wetland is located in the middle of China, at the junction of Henan, Shanxi, and Shaanxi provinces (1), with a total area of 28,500 hectares, accounting for 42% of the entire reserve area. The altitude is between 300 and 1,500 m. The average temperature in Sanmenxia in January is −5~2°C. Whooper swans live in the Sanmenxia Swan National Wetland Park from the end of October to the end of March each year. Whooper swans mainly live in shallow waters with open water, sufficient food, and a safe habitat.

Rongcheng Swan Lake National Nature Reserve is located in Chengshan Town, Rongcheng City, the easternmost part of Shandong Province. It is mainly composed of Yuehu Lake, Fish Farm Bay, Linluo Bay, and surrounding mountains, with a total area of 1,675 hectares, including the core area 668 hectares, buffer zone 628 hectares, and experimental area 379 hectares, with an average elevation of 7 m. The average temperature in Rongcheng in January is −5~1°C.

### Sample Collection

All fecal samples were collected from wild whooper swans in Sanmenxia, Henan, and Rongcheng, Shandong. Ten sampling sites were identified based on the distribution of wintering swans in the study area and the accessibility of their terrestrial resting places. Three parallel transects were established at each sampling site, covering most parts of their terrestrial resting area. Transect length differed, depending on the topography of the area (i.e., 100–500 m). Transects were walked on foot, with one observer on each transect line. Five fresh and clean fecal samples were collected at random along transect lines from two sites. In order to ensure the uniformity of sample time, all samples were collected in January. The collected fresh samples were stored in a mobile refrigerator, transported back to the laboratory, and stored in a refrigerator at −80°C.

### DNA Extraction, Purification, and 16S rRNA Gene Sequencing

The total genomic DNA of the fecal flora was purified with a fecal DNA extraction kit (QIAamp DNA Stool Mini Kit; QIAGEN, Hilden, Germany) according to the manufacturer's protocol. The integrity of extracted genomic DNA was verified by 1.0% agarose gel electrophoresis. When the electrophoretic band showed a single bright band without significant dragging, it indicated that the genome was intact without degradation. The concentration and purity of genomic DNA were determined using Qubit dsDNA HS Assay Kit (Life Technologies, Carlsbad, CA, USA). The extracted genomic DNA was stored in a −80°C freezer for subsequent polymerase chain reaction (PCR) and sequencing.

The V3–V4 hypervariable regions of bacterial 16S rRNA gene was amplified using primers 338F (5′-ACTCCTACGGGAGGCAGCA-3′) and 806R (5′-GGACTACHVGGGTWTCTAAT-3′). The PCR volume was 50 ml containing 10 ml of PCR buffer, 0.2 μl of High-Fidelity DNA Polymerase, 1 μl of dNTP, 10 μl of GC Enhancer, 1.5 μl each of 10 μM forward and reverse primers, and 60 ng of template DNA; and the rest of the volume was DNase-free sterile water. The PCR conditions were as follows: 95°C for 5 min, followed by 25 cycles of 95°C for 30 s, 50°C for 30 s, 72°C for 40 s, and 72°C for 7 min. The PCR products were purified with DNA gel extraction kit (AxyGen, Shanghai, China). Ultimately, high-throughput sequencing on an Illumina HiSeq 2500 platform (Illumina Inc., San Diego, CA, USA) was conducted at Biomarker Technologies Corporation (Beijing, China).

The raw image data files obtained by high-throughput sequencing were transformed into the original sequence reads by Base Calling analysis, and the results were stored in FASTQ (fq) file format, including the sequence information of the sequenced reads and its corresponding sequencing quality information.

### Statistical Analysis

The program PRINSEQ (21) was used for quality filtration. All sequences were grouped into operational taxonomic units (OTUs) with a 97% sequence similarity level using the UCLUST program against the SILVA database.

Alpha diversity indices (ACE, Chao1, Shannon, and Simpson) were presented as the means ± SD and were calculated by QIIME 2 from rarefied samples using for richness and diversity indices of the bacterial community. The ACE and Chao indices were used to estimate the number of OTUs in samples and are commonly used in ecology to estimate the total number of species. Shannon's index and Simpson's diversity index are common measures of diversity, which reflect richness and evenness of the samples. Non-metric multidimensional scaling (NMDS) based on the unweighted UniFrac distance matrices was performed to determine beta diversity. A one-way analysis of similarity (ANOSIM) was performed to determine the differences in bacterial communities among groups (22).

Linear discriminant analysis (LDA) effect size (LEfSe) analysis was performed to reveal the significant ranking of abundant modules in two sites samples (23). A size-effect threshold of 4.0 on the logarithmic LDA score was used for discriminative functional biomarkers.

The raw sequences obtained in this study have been submitted to the National Center for Biotechnology Information (NCBI) Sequence Read Archive (accession number PRJNA721779).

## Results

### Sequence Statistics

A total of 679,728 effective sequences were obtained from 10 fecal samples of two study sites; and 53,408 to 72,897 (mean 67,973 ± 5,748) effective sequence (mean length 423 bp) were obtained from each sample. A total of 481 OTUs were obtained at a sequence-similarity level of 97%, with 238 ± 58 (range: 143–319) as the mean number of OTUs per sample.

**Figure 2:**
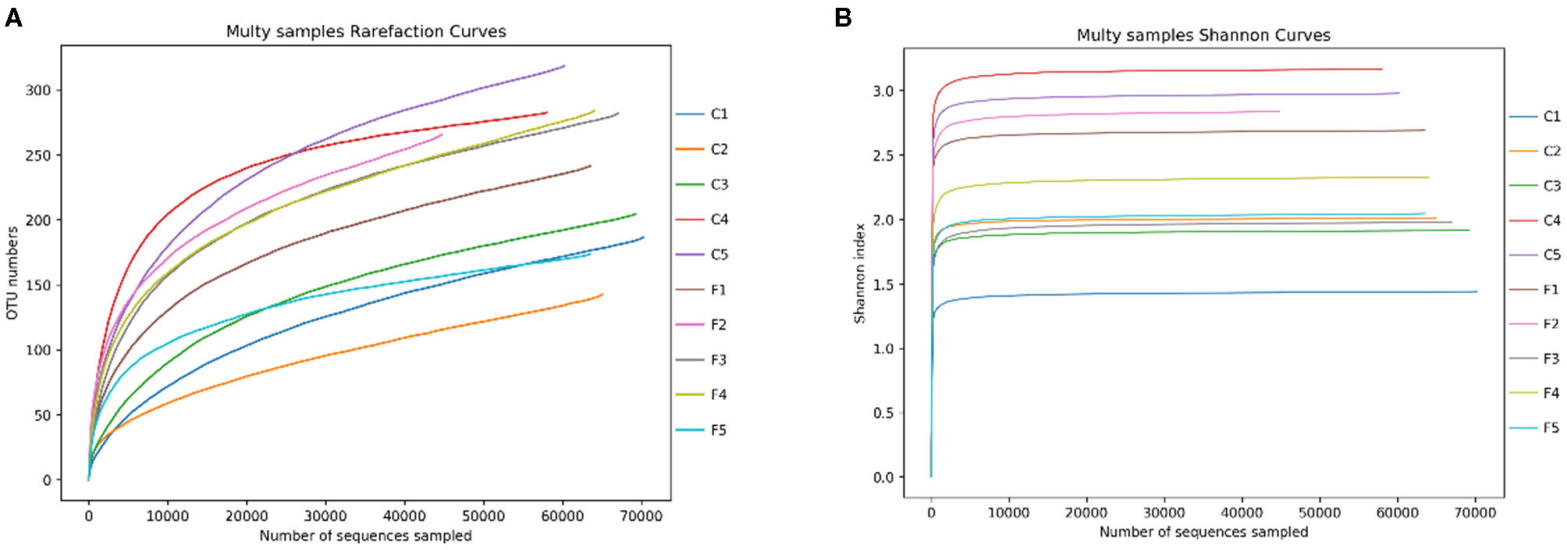
The rarefaction curves of OTUs **(A)** and Shannon index for the 10 samples **(B)**. C1–C5 represent the samples collected in Sanmenxia, and F1–F5 represent the samples collected in Rongcheng.

These OTUs were classified into 17 phyla, 32 classes, 69 orders, 133 families, 285 genera, and 320 species. The rarefaction curve showed that increasing the sequencing depth can obtain more OTUs (Figure 2A), while the sparse curve of Shannon's index showed that the bacterial diversity of all samples has reached a plateau, and deeper sequencing has no significant impact on diversity (Figure 2B).

**Figure 3:**
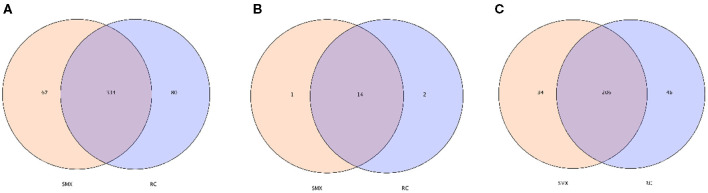
Venn diagram. The Venn diagrams show the numbers of OTUs (97% sequence identity) **(A)**, phyla **(B)** and genera **(C)** that were shared or not shared by Sanmenxia(SMX) and Rongcheng(RC) individuals depending of overlaps (Sanmenxia Swan National Wetland Park in Henan: SMX; Swan Lake National Nature Reserve in Shandong Rongcheng: RC).

In the OTU level, there are 334 OTUs shared by whooper swan individuals of two sites. The number of phyla and genera shared by two sites was 14 and 206, respectively (Figure 3).

### Relative Abundance and Core Microbiota

**Figure 4:**
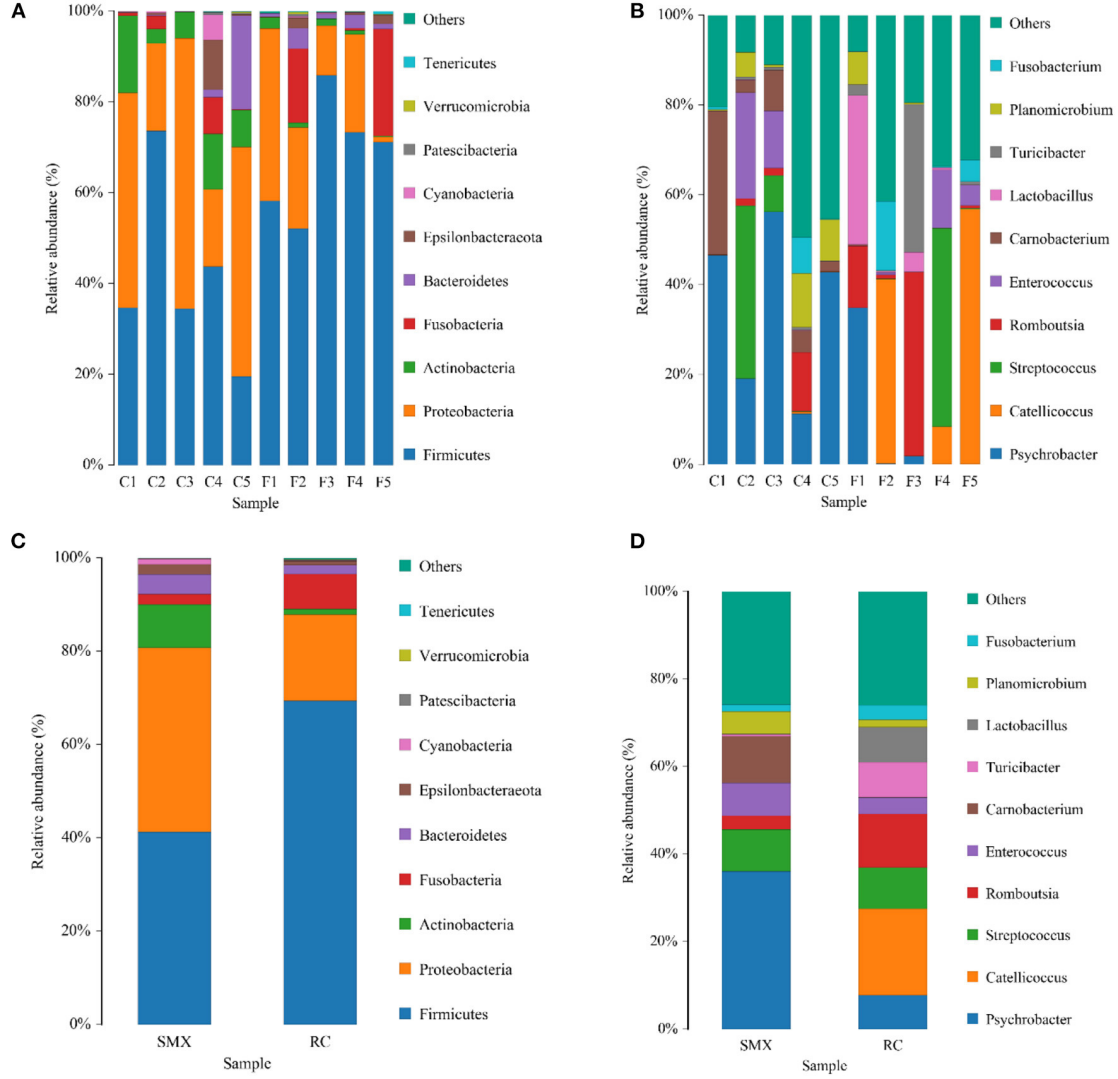
Bar chart of relative abundance. Relative abundance (%) of the 10 most abundant bacteria phyla (**A** for individuals and **C** for groups) and genera (**B** for individuals and **D** for groups) obtained from 10 fecal samples of whooper swans at two study sites. Others, Bacteria taxa with ≤1% abundance; Unclassified, sequences that could not be classified (SMX: C1–C5; RC: F1–F5).

The top 10 phyla and the top 10 genera according to relative abundance of the fecal bacteria that were present in SMX (Sanmenxia) and RC (Rongcheng) samples are displayed in Figure 4.

At the level of phylum, the SMX and RC microbiota were dominated by Firmicutes and Proteobacteria, followed by Actinobacteria, Fusobacteria, and Bacteroidetes. The dominant genera of SMX were *Psychrobacter, Carnobacterium, Streptococcus*, and *Enterococcus*. The dominant genera of RC were *Catellicoccus, Romboutsia, Streptococcus, Lactobacillus, Turicibacter*, and *Psychrobacter*.

### Alpha Diversity and Beta Diversity Analyses

The alpha diversity analysis showed no significant differences in ACE (288.36 ± 45.42 and 313.68 ± 78.20), Chao 1 (271.28 ± 61.49 and 302.76 ± 65.89), Simpson (0.21 ± 0.11 and 0.21 ± 0.07), Shannon (2.30 ± 0.74 and 2.38 ± 0.38) indices between SMX and RC (*p* > 0.05).

**Figure 5:**
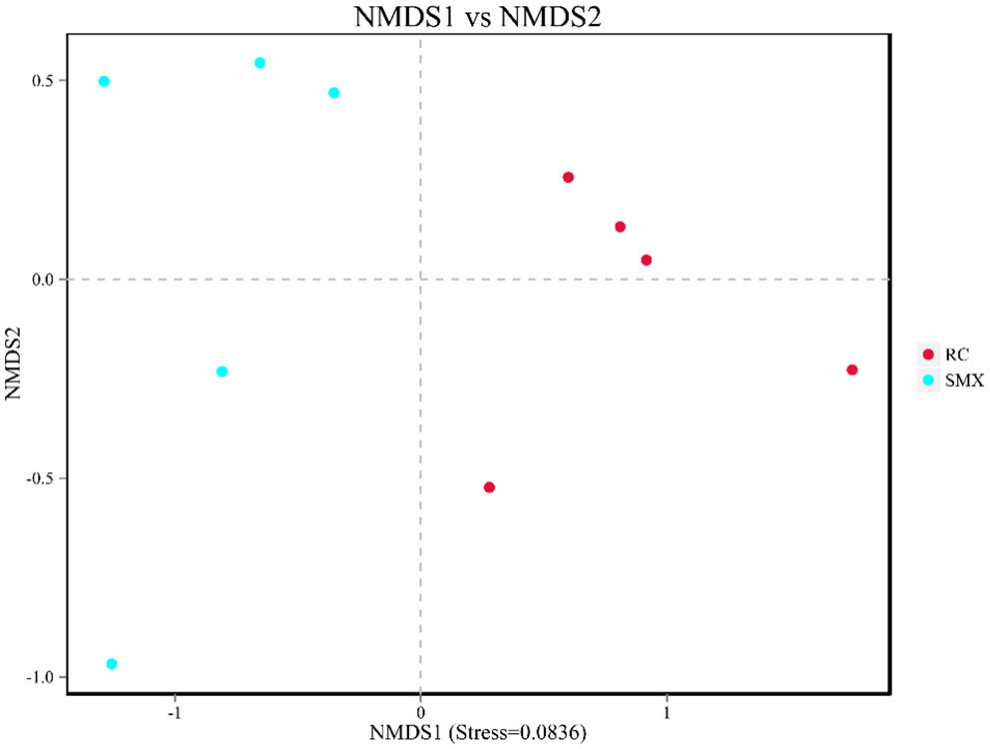
Non-metric multidimensional scaling (NMDS) analysis. Each point in the graph represents one sample, and different colors represent different groups. The distance between points represents the level of difference. Stress lower than 0.2 indicates that the NMDS analysis is reliable. The closer the samples in the graph, the higher their similarity.

**Figure 6:**
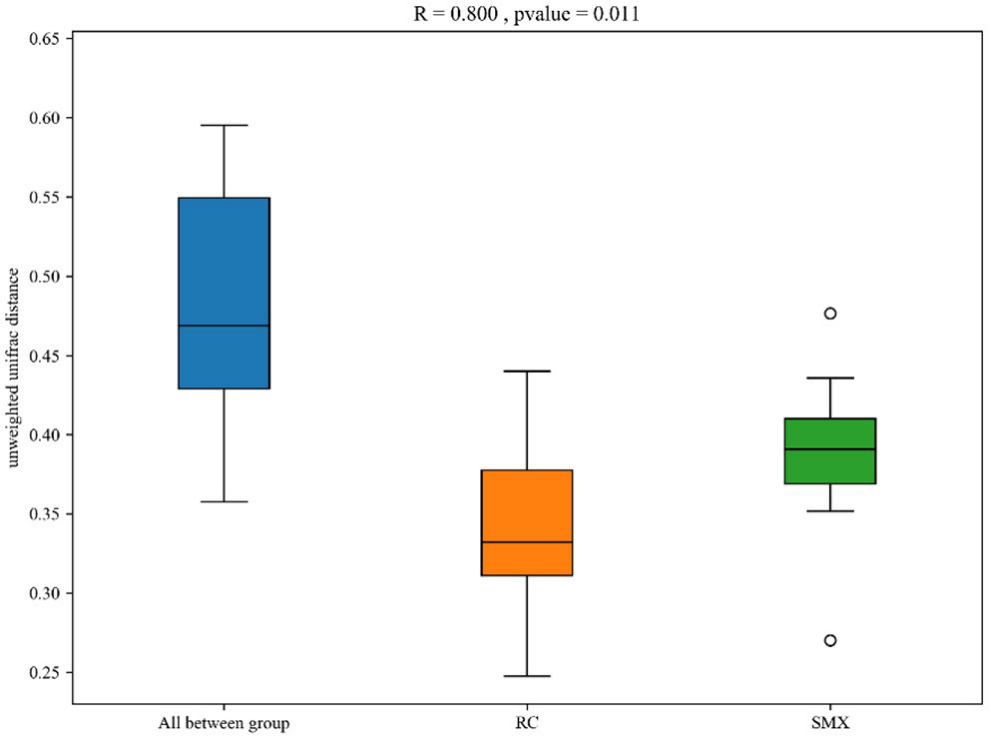
Box plot of inter-group and intra-group beta distance [analysis of similarity (ANOSIM)]. *R*-value range (0–1): *R*-values close to 0 represent no significant differences between inter-group and intra-group; R-values close to 1 show that inter-group differences are greater than intra-group differences. Boxes represent the interquartile range (IQR; between the 25th and 75th percentiles); the horizontal line inside the box defines the median, and outliers >1.5 and <3 times the IQR.

Beta diversity was used to determine whether there was a difference in bacterial community compositions between Sanmenxia and Rongcheng groups. The NMDS plot based on unweighted UniFrac distance showed the dissimilarity of microbial community and revealed a distinct structure between swans from two sites (Figure 5). Inter-group and intra-group beta distance is shown in the box plot, with the results showing significant differences in bacterial communities between Sanmenxia and Rongcheng groups (ANOSIM: R = 0.80, *p* = 0.011) (Figure 6).

### Significant Difference Analysis of Different Sites

**Figure 7:**
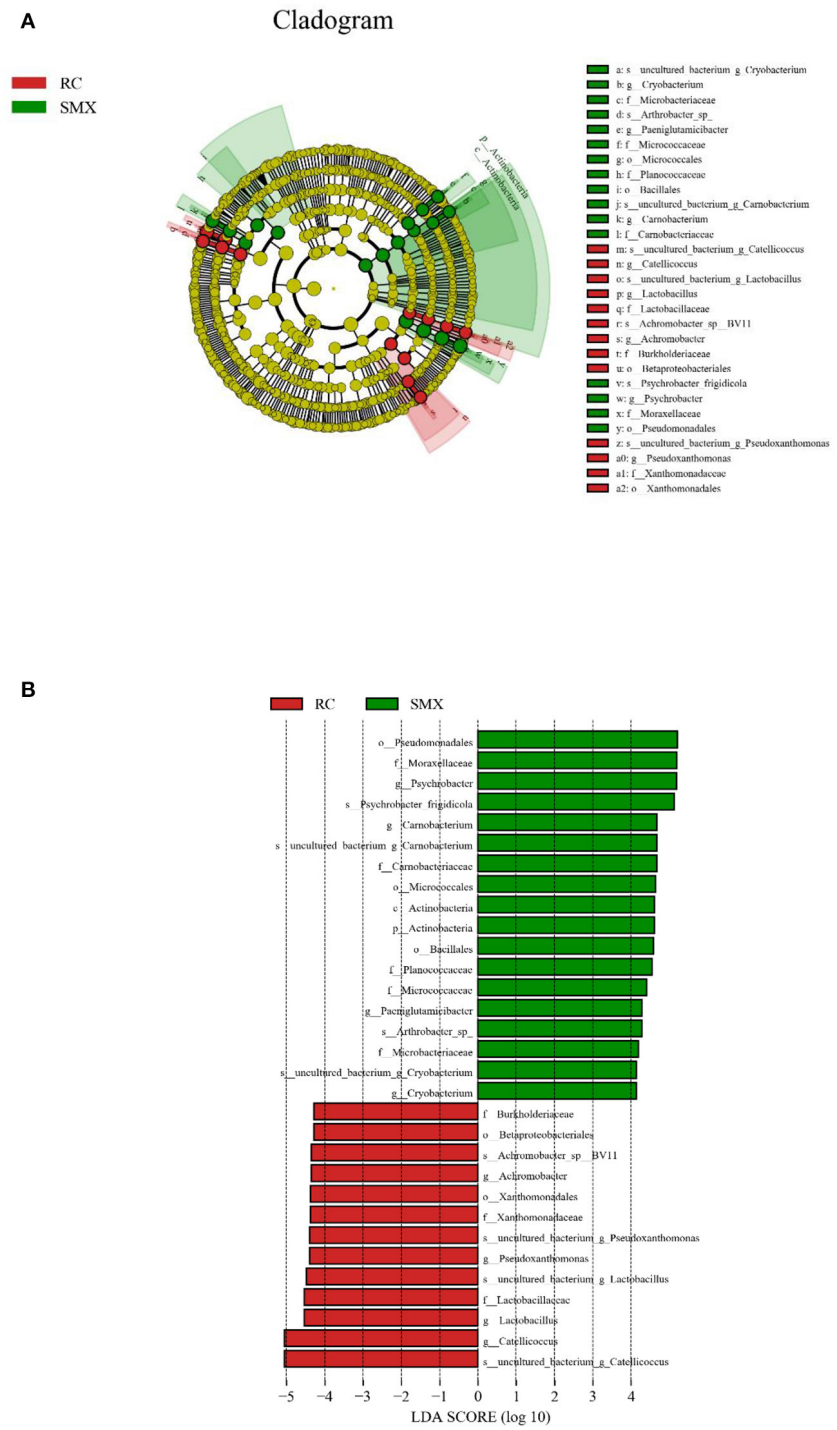
Linear discriminant analysis effect size (LEfSe) analysis. **(A)** The cladogram diagram shows the microbial species with significant differences in the two groups. Red and green indicate different groups, with the species classification at the level of phylum, class, order, family, and genus shown from the inside to the outside. **(B)** Plot from LEfSe analysis. The plot was generated using the online LEfSe project. The length of the bar column represents the linear discriminant analysis (LDA) score. The figure shows the microbial taxa with significant differences between Sanmenxia and Rongcheng (LDA score > 4.0).

LEfSe analysis was performed to reveal the significant ranking of abundant modules. The cladogram showed differences in 31 taxa among two wintering sites (Figure 7A). The plot from LEfSe analysis (Figure 7B) displays LDA scores of microbial taxa with significant differences of the two sites. On the phylum level, the biomarker demonstrating significant differences between two sites was Actinobacteria (LDA > 4.0, *p* < 0.05). On the genus level, the biomarkers demonstrating significant differences between two sites were *Psychrobacter, Catellicoccus, Carnobacterium, Lactobacillus, Pseudoxanthomonas, Achromobacter, Paeniglutamicibacter*, and *Cryobacterium* (LDA > 4.0, *p* < 0.05).

## Discussion

Henan Sanmenxia and Shandong Rongcheng are important wintering places for whooper swans in China. There is no related research on the intestinal microbiota of whooper swans of different wintering places. The bacterial microbiota plays an important role in nutrition and energy metabolism, immune homeostasis, and reproduction (24). The habitat environments of the two wintering areas are different, and studies have shown that diet nutrition and habitat environment are both potential driving factors of animal gut microbial communities (25–28). In this study, the Illumina HiSeq platform was used to study the differences in the bacterial microbiota of whooper swans in different locations based on 16S rRNA gene high-throughput sequencing.

In terms of alpha diversity, the richness and diversity of the observed OTUs showed no difference between the swans of Sanmenxia and Rongcheng. In terms of intestinal microbiota composition, NMDS analysis and ANOSIM s demonstrated that there were significant differences in intestinal microbiota composition between the two site groups.

In this study, it was found that Firmicutes and Proteobacteria were the core microbiota of two wintering whooper swans, and the sum of the relative abundance of the two phylum bacteria was higher than 80%. Previous studies have found that Firmicutes and Proteobacteria were also the dominant microbiota of other birds, with relatively high abundance, such as *Anser cygnoides* (29), *Amazona farinosa, Ara ararauna, Ara chloropterus* (30), *Strigops habroptilus* (31), *Pelecanoides urinatrix* (33), and *Anser indicus* (32). Firmicutes, as an important metabolic phylum in the intestine, can decompose compound sugars, polysaccharides, and fatty acids in foods and produce energy and nutrients for animals to absorb and utilize. According to previous studies, Proteobacteria have a variety of physiological functions, can utilize a large amount of carbon sources, and play an important role in the host's energy accumulation (34–36).

In addition, at the level of phylum, other dominant bacteria phyla include Actinobacteria, Fusobacteria, and Bacteroidetes. Fusobacteria is a kind of strict obligate anaerobic bacteria with negative Gram stain and high nutritional requirements. Fusobacteria has also been found in other birds, such as Adelie penguin (*Pygoscelis adeliae*) (37), emu (*Dromaius novaehollandiae*) (38), and vulture (*Aegypius monachus*) (39). The Fusobacteria can produce butyrate, which in turn promotes fat accumulation in the body and enhances immunity (40). The average proportion of Bacteroidetes in the intestinal microbiota of whooper swans in Sanmenxia and Rongcheng was 4.25 and 1.90%, respectively. Compared with human intestinal microbiota, the relative abundance of Bacteroidetes was lower. Bacteroidetes is core microbiota of human and has many functions involved in carbohydrate metabolism, steroid and bile acid metabolism, and carbohydrate fermentation (41). An increase in the ratio of Firmicutes and *Bacteroides* (F/B) will lead to changes in the body's ability to metabolize fat and increase the ability of the intestine to absorb nutrients and transform energy (42, 43). This study found that the F/B (36.5) of Rongcheng whooper swan was higher than that of Sanmenxia whooper swan (9.71), which indicated that the function of the intestine microbiota of Rongcheng whooper swan might be more inclined to body fat metabolism, so that the host can obtain more from energy from food. A high proportion of Firmicutes and *Bacteroides* can significantly inhibit enteropathogenic bacteria (44).

LEfSe analysis showed that at the phylum level, the relative abundance of Actinobacteria was significantly higher in Sanmenxia swans than Rongcheng swans. This may be due to the abundant fiber-rich plant food in Sanmenxia. The relative abundance of Actinobacteria is positively correlated with host fiber intake (45). Actinobacteria are ubiquitous in aquatic and terrestrial ecosystems. They are important bacteria that produce secondary metabolites such as enzymes and antibiotics. At the same time, they produce secondary metabolites in the intestines of animals and are used as powerful antibiotics in health or disease. It has an important role (46, 47), and it exists widely in various animals (34, 48, 49).

At the genus level, the relative abundance of *Psychrobacter* and *Carnobacterium* in Sanmenxia was significantly higher in Rongcheng, while the relative abundance of *Catellicoccus* and *Lactobacillus* was significantly higher in Rongcheng than in Sanmenxia swans. *Psychrobacter* often exist in fish and shrimp (50–52), and *Carnobacterium* are spoilage bacteria in fish food (53). These may show that small fish, shrimps, mollusks, etc. are the food of whooper swans in the wintering sites; and the whooper swans may have more of this kind of food in Sanmenxia. This is somewhat different from the previous studies on the feeding habits of whooper swans. Previous studies mostly used the method of plant microscope comparison and would ignore some animal food (7, 13). The genera *Catellicoccus* is composed of a single species, viz., *Catellicoccus marimammalium*. *Catellicoccus* cells are arranged in pairs or chains and are Gram-positive, non-motile, non-spore-forming and generally coccoid. Based on 16S rRNA gene sequence studies, *Catellicoccus, Melissococcus*, and *Pilibacter* form a distinct branch, related to the family Enterococcaceae (54). *C. marimammalium* was present in gull fecal samples collected in North America, Bradford Beach (Milwaukee, WI), and Grant Park (South Milwaukee, WI) (55, 56). Targeting this bacterial species might be useful for detecting fecal contamination in waterfowl-impacted waters. *Lactobacillus* is the main normal microbiota in human and animal stomachs and small intestines, which can inhibit the reproduction of pathogenic bacteria (57).

*Streptococcus* was detected in the samples and is potentially pathogenic. Birds and poultry are susceptible to *Streptococcus*. The source and route of infection of *Streptococcus* are usually unknown, although the carrier state can be maintained for several months (58). Whooper swans are migratory birds and take regular long-distance flights every year. They may be exposed to potential pathogenic microbiota at multiple stops. In addition, during the migration, lifestyle changes and dietary selective pressure may destroy the stable intestinal microbiota, which will lead to physiological stress, can cause a decline in immune function, and be more susceptible to pathogenic bacteria (59).

The studies of bird intestinal pathogens are of great value for understanding the health status of birds, especially the conservation of endangered species. Whooper swan is a migratory bird, and death cases of whooper swan avian influenza occur every year in China, indicating that it poses a threat to public health security. Whooper swans in Sanmenxia and Rongcheng have a large overlap with other birds, and even some poultry and livestock habitats. A large number of migrating birds gather every winter, and intestinal pathogen is easily transmitted between birds, poultry, and livestock through feces. This reminds us that while protecting whooper swans, we must pay lots of attention on the defense of human and zoonotic diseases. In the follow study, we plan to do some integrated research about the intestinal pathogenic bacteria, immunoglobulins, and avian influenza of migratory whooper swans for protecting the whooper swan population and strengthening the public health security. In addition, seagrass is an important food resource for wintering whooper swans in Rongcheng. However, pollution has caused changes in the quality of seawater and reduced aquatic organisms such as large leaf algae. It is recommended to rationally plan the scale and layout of the development of mariculture for improving the function of wetlands and protecting the wintering habitat of whooper swans.

This study analyzed the differences in the gut microbiota of swans in different wintering places in China and also proved that different wintering places and dietary environment will affect the composition of the gut microbiota of swans. Due to the limitation of sample collection and experimental conditions, it is really difficult to conduct a specific analysis of the influence coefficient of whooper swan food resources on the gut microbiota. In the following period, we will analyze the characteristics of the gut microbiota between captive population and the wild population, and in different wintering periods for more interesting facts.

## Data Availability Statement

The data presented in the study are deposited in the NCBI Sequence Read Archive repository, accession number PRJNA721779.

## Ethics Statement

The animal study was reviewed and approved by the Ethics Committee of Forest Ecology, Environment and Protection, Chinese Academy of Forestry.

## Author Contributions

WW, LY, and GZ designed the study, performed the experiments, and collected the fecal samples. WW, LY, and SH analyzed the data and wrote the manuscript. All authors contributed to the article and approved the submitted version.

## Conflict of Interest

The authors declare that the research was conducted in the absence of any commercial or financial relationships that could be construed as a potential conflict of interest.
